# Tobacco

**Published:** 1877-10-20

**Authors:** 


TOBACCO.—We are not an advocate of the Tobacco habit, but as nearly every body uses it, and are likely to do so in the face of all the lectures which are made against it, and among them, no doubt, nine-tenths of our Medical readers, we wish to say to them that the evil results of tobacco may in a great measure be avoided by using a pure and properly manufactured article, and such we have found to be *the genuine Durham Smoking Tobacco.* Doctors may safely advise their friends to use this as probably the purest, least injurious article. See advertisement.

